# IL1RAPL1 Associated with Mental Retardation and Autism Regulates the Formation and Stabilization of Glutamatergic Synapses of Cortical Neurons through RhoA Signaling Pathway

**DOI:** 10.1371/journal.pone.0066254

**Published:** 2013-06-13

**Authors:** Takashi Hayashi, Tomoyuki Yoshida, Moonjin Ra, Ryo Taguchi, Masayoshi Mishina

**Affiliations:** 1 Department of Molecular Neurobiology and Pharmacology, Graduate School of Medicine, University of Tokyo, Tokyo, Japan; 2 Department of Metabolome, Graduate School of Medicine, University of Tokyo, Tokyo, Japan; 3 PRESTO, Japan Science and Technology Agency, Kawaguchi, Saitama, Japan; 4 Brain Science Laboratory, The Research Organization of Science and Technology, Ritsumeikan University, Kusatsu, Shiga, Japan; Georgia Regents University, United States of America

## Abstract

Interleukin-1 receptor accessory protein-like 1 (IL1RAPL1) is associated with X-linked mental retardation and autism spectrum disorder. We found that IL1RAPL1 regulates synapse formation of cortical neurons. To investigate how IL1RAPL1 controls synapse formation, we here screened IL1RAPL1-interacting proteins by affinity chromatography and mass spectroscopy. IL1RAPL1 interacted with Mcf2-like (Mcf2l), a Rho guanine nucleotide exchange factor, through the cytoplasmic Toll/IL-1 receptor domain. Knockdown of endogenous Mcf2l and treatment with an inhibitor of Rho-associated protein kinase (ROCK), the downstream kinase of RhoA, suppressed IL1RAPL1-induced excitatory synapse formation of cortical neurons. Furthermore, we found that the expression of IL1RAPL1 affected the turnover of AMPA receptor subunits. Insertion of GluA1-containing AMPA receptors to the cell surface was decreased, whereas that of AMPA receptors composed of GluA2/3 was enhanced. Mcf2l knockdown and ROCK inhibitor treatment diminished the IL1RAPL1-induced changes of AMPA receptor subunit insertions. Our results suggest that Mcf2l-RhoA-ROCK signaling pathway mediates IL1RAPL1-dependent formation and stabilization of glutamatergic synapses of cortical neurons.

## Introduction

Interleukin-1 receptor accessory protein-like 1 (IL1RAPL1) is associated with mental retardation (MR) and autism spectrum disorder (ASD) [1], [2]. MR and ASD are highly heterogenous neurodevelopmental disorders. MR, defined as a failure to develop cognitive abilities, is the most frequent cause of serious handicap in children and young adults [3], while ASDs are characterized by severe deficits in socialization, impaired communication, and a limited range of interests and behavior [4], [5]. The observation that mutations in the gene encoding IL1RAPL1 may lead to MR, ASD or both is in line with recent studies noting overlap of genetic loci in susceptibility to these disorders [6]–[10]. In fact, cognitive impairment is common in autism, and ∼70% of autistic individuals suffer from MR [11]. Although the underlying causes of these mental disorders are extremely heterogeneous, molecular alterations in monogenic disorders may identify common pathogenic pathways shared by MR or ASD or both [12]. We found that presynaptic IL1RAPL1 controls *in vivo* synapse formation of olfactory sensory neurons of zebrafish [13]. In mouse cortical neurons, postsynaptic IL1RAPL1 mediates excitatory synapse formation through *trans*-synaptic interaction with presynaptic protein tyrosine phosphatase (PTP) δ [14]. We thus proposed that the impairment of synapse formation underlies certain forms of MR and ASD as a common pathogenic pathway shared by these mental disorders [14]. Then, the question arises how IL1RAPL1 regulates synapse formation.

IL1RAPL1 is a member of the IL-1/Toll receptor family, but has no activity to mediate immune signals as a component of the receptors for IL-1 family cytokines [15], [16]. In contrast to the members of the IL-1 receptor family mediating immune responses to IL-1 family cytokines, IL1RAPL1 is selectively expressed in the brain. IL1RAPL1 contains three extracellular immunoglobulin (Ig)-like domains, a transmembrane domain and an intracellular Toll/IL-1 receptor (TIR) domain similar to other IL-1 receptor family proteins. IL1RAPL1 stimulates the increase of dendritic protrusions through the TIR domain [14]. To investigate how IL1RAPL1 controls the number of dendritic protrusions and synapse maturation, we here isolated the molecules interacting with the cytoplasmic domain of IL1RAPL1 by affinity chromatography. Among several molecules identified, we focused on Mcf2-like (Mcf2l), a Rho guanine nucleotide exchange factor (GEF). Studies with siRNA against Mcf2l and a Rho-associated protein kinase (ROCK) inhibitor suggest that Mcf2l-RhoA-ROCK signaling pathway mediates IL1RAPL1-dependent formation and stabilization of glutamatergic synapses between cortical neurons.

## Results

**Figure 1:**
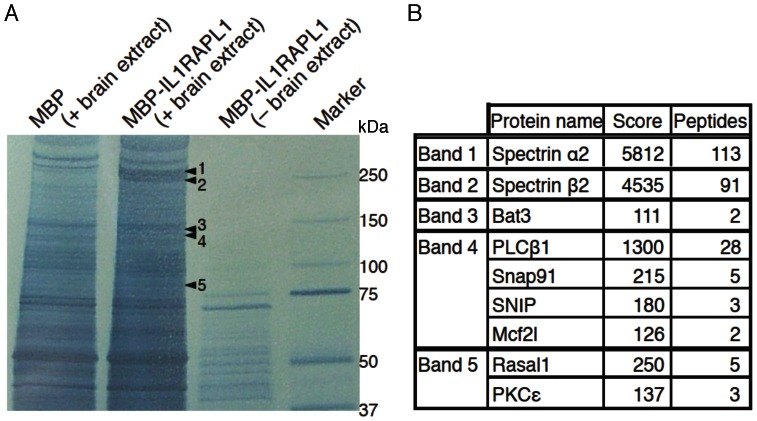
Identification of IL1RAPL1-ICD-binding proteins by affinity chromatography. **A,** Negative staining of IL1RAPL1-binding proteins from the brain extracts, resolved by SDS-PAGE. Affinity chromatography of brain extracts was performed with maltose binding protein (MBP) conjugated with or without the cytoplasmic domain of IL1RAPL1. Protein bands specific to or thicker on the “MBP-IL1RAPL1 (+ brain extract)” lane (arrowheads 1–5) were excised and analyzed by LC-MS/MS. After subtraction of proteins detected in the control “MBP (+ brain extract)” lane, 9 candidate interactors were identified. **B,** List of identified proteins from each gel band. Numbers of identified peptides for each protein and scores of Mascot searches are shown.

To investigate the postsynaptic signaling mechanism of synapse formation by IL1RAPL1, we first isolated proteins interacting with the intracellular domain (ICD) of IL1RAPL1 from mouse forebrain by affinity chromatography. Total mouse forebrain proteins were extracted in 2M NaCl and the extract was loaded onto affinity columns of amylose resins coupled to IL1RAPL1-ICD fused with maltose-binding protein (MBP) or to MBP as a control. Proteins bound to IL1RAPL1-ICD-MBP or MBP beads were eluted in 0.5 M NaCl and separated by SDS-PAGE (Figure 1A). We found five bands with much stronger staining intensities in the eluate from IL1RAPL1-ICD-MBP beads than in the eluate from MBP beads. Analysis of proteins in the five bands by liquid chromatography-tandem mass spectrometry (LC-MS/MS) identified spectrin α2 in band #1, spectrin β2 in band #2, Bat3 in band #3, phospholipase C β1 (PLCβ1), Snap91, Snap-25-interacting protein (SNIP) and MCF.2 cell line derived transforming sequence-like (Mcf2l) in band #4, Ras protein activator like 1 (Rasal1) and protein kinase C ε (PKCε) in band #5 as candidates for IL1RAPL1-ICD binding proteins (Figure 1B).

**Figure 2:**
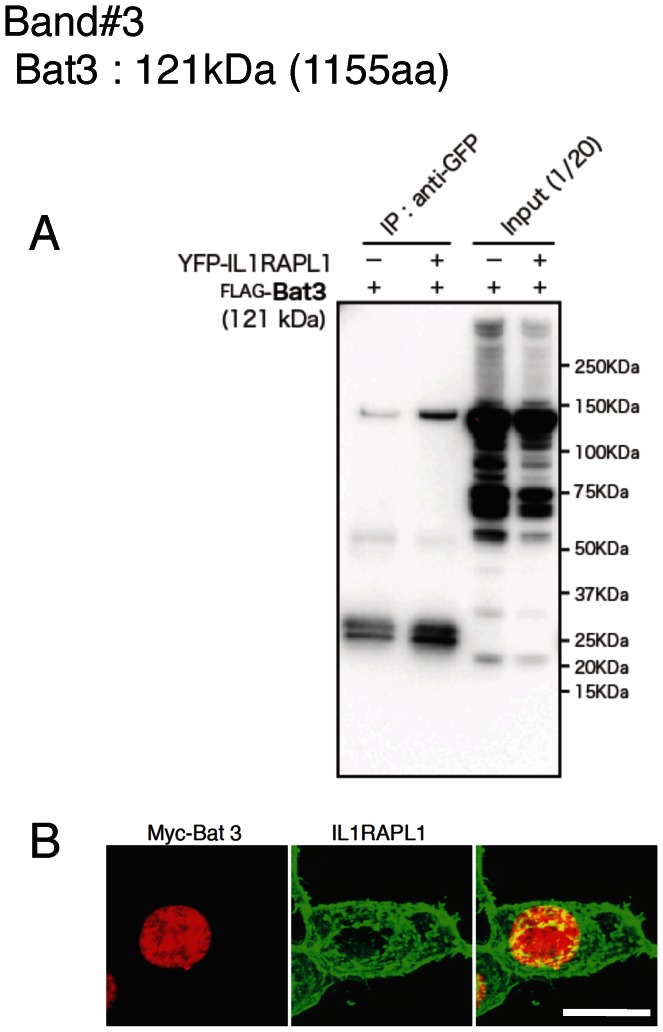
Analysis of IL1RAPL1-binding protein candidates (band #3). **A,** Coimmunoprecipitation of YFP-IL1RAPL1 with FLAG-Bat3 in HEK 293T cells. Immunoprecipitation with anti-GFP antibody and total cell lysates, followed by western blotting with anti-FLAG antibody are shown. **B,** Colocalization of IL1RAPL1 (middle, green) and myc-Bat3 (left, red) in HEK 293T cells. Merged images are shown (right). Scale bar, 10 μm.

**Figure 3:**
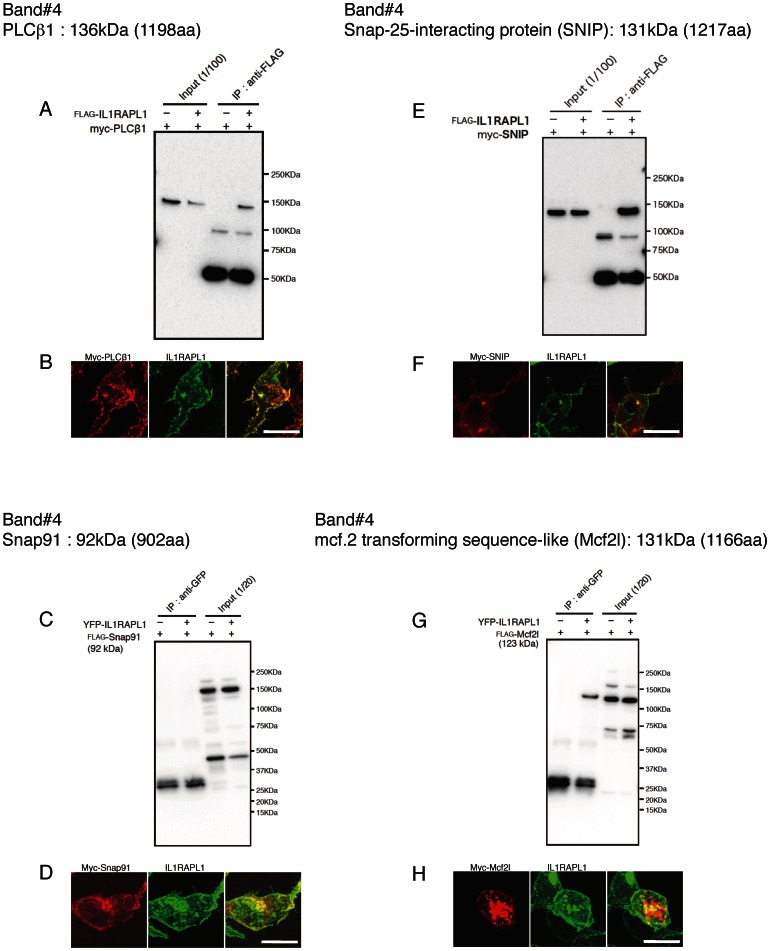
Analysis of IL1RAPL1-binding protein candidates (band #4). **A, C, E, G,** Coimmunoprecipitation of FLAG-IL1RAPL1 with myc-PLCβ1 (**A**) and myc-SNIP (**E**) and that of YFP-IL1RAPL1 with FLAG-Snap91 (**C**) and FLAG-Mcf2l (**G**) in HEK 293T cells. Immunoprecipitation with anti-FLAG antibody followed by western blotting with anti-Myc antibody (**A**, **E**) and that with anti-GFP antibody followed by western blotting with anti-FLAG antibody (**C**, **G**) are shown. **B, D, F, H,** Colocalization of IL1RAPL1 (middle, green) and myc-PLCβ1 (**B**), myc-Snap91 (**D**), myc-SNIP (**F**) and myc-Mcf2l (**H**) (left, red) in HEK 293T cells. Merged images are shown (right). Scale bar, 10 μm.

**Figure 4:**
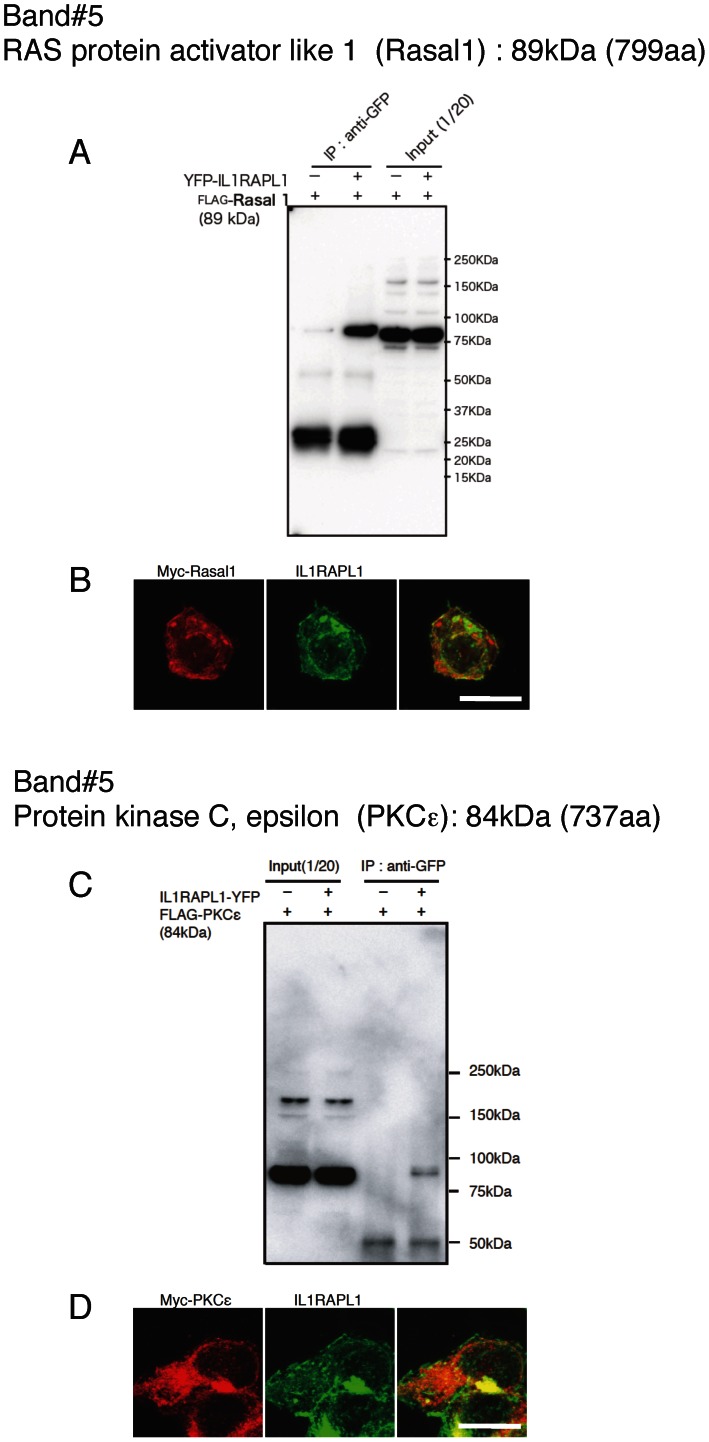
Analysis of IL1RAPL1-binding protein candidates (band #5). **A, C,** Coimmunoprecipitation of YFP-IL1RAPL1 with FLAG-Rasal1 (**A**) and FLAG-PKCε (**C**) in HEK 293T cells. Immunoprecipitation with anti-GFP antibody followed by western blotting with anti-FLAG antibody is shown. **B, D,** Colocalization of IL1RAPL1 (middle, green) and myc-Rasal1 (**B**) and myc-PKCε (**D**) (left, red) in HEK 293T cells. Merged images are shown (right). Scale bar, 10 μm.

We expressed these candidate proteins tagged with FLAG or myc in HEK 293T cells together with IL1RAPL1 or IL1RAPL1 tagged with YFP or FLAG to examine their interactions except for spectrin α2 and β2. Anti-GFP antibody immunoprecipitated FLAG-Bat3 together with YFP-IL1RAPL1, but the antibody precipitated FLAG-Bat3 in the absence of YFP-IL1RAPL1 though the amount was smaller (Figure 2A). Immunocytochemistry showed that myc-Bat3 was localized in the nucleus (Figure 2B) as reported [17]. Thus, the interaction between Bat3 and IL1RAPL1, if any, may not be physiological. Anti-FLAG antibody immunoprecipitated myc-PLCβ1 and myc-SNIP together with FLAG-IL1RAPL1 (Figure 3A and 3E). Anti-GFP antibody immunoprecipitated FLAG-Mcf2l together with YFP-IL1RAPL1 (Figure 3G). Consistently, myc-PLCβ1, myc-SNIP and myc-Mcf2l were colocalized with IL1RAPL1 (Figure 3B, 3F and 3H). On the other hand, anti-GFP antibody failed to immunoprecipitate FLAG-Snap91 together with YFP-IL1RAPL1 (Figure 3C), although myc-Snap91 and IL1RAPL1 appeared to be colocalized (Figure 3D). Anti-GFP antibody immunoprecipitated FLAG-Rasal1 and FLAG-PKCε together with YFP-IL1RAPL1 (Figure 4A and 4C). Consistently, myc-Rasal1 and myc-PKCε were colocalized with IL1RAPL1 (Figure 4B and 4D). These results suggest that IL1RAPL1 can interact with PLCβ1, SNIP, Mcf2l, Rasal1 and PKCε.

**Figure 5:**
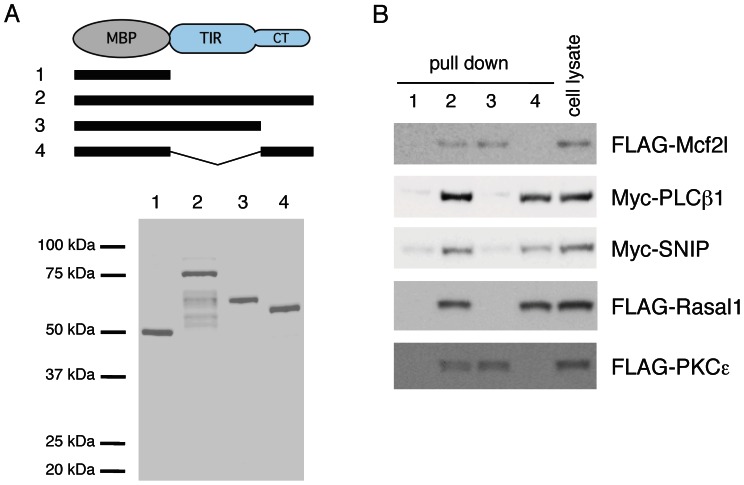
Pull down experiments of each IL1RAPL1 intracellular domain with interacting proteins. **A,** Schematic structures (top) and SDS-PAGE followed by Coomassie Brilliant Blue staining (bottom) of purified MBP (1) and MBP-fusion proteins with the whole cytoplasmic region (2), the TIR domain (3) and the CT domain (4) of IL1RAPL1. **B,** Cell lysates from HEK 293T cells transfected with FLAG-Mcf2l, myc-PLCβ1, myc-SNIP, FLAG-Rasal1 or FLAG-PKCε were incubated with amylose resins coupled to MBP (1) or MBP-fusion protein with the whole cytoplasmic region (2), the TIR domain (3) or the CT domain (4) of IL1RAPL1. Precipitates were analyzed by SDS-PAGE followed by immunoblotting with anti-FLAG or anti-Myc antibody.

To identify the binding site of these proteins, we generated the TIR and CT domains of IL1RAPL1 fused with MBP (Figure 5A). Pull-down experiments showed that FLAG-Mcf2l and FLAG-PKCε interacted with the TIR domain of IL1RAPL1, whereas myc-PLCβ1, myc-SNIP and FLAG-Rasal1 bound to the CT domain (Figure 5B). Since IL1RAPL1 stimulates the increase of dendritic protrusions through the TIR domain [14], Mcf2l and PKCε are candidate mediators of the spinogenic signaling through IL1RAPL1. It is known that PKCε interacts with the TIR domains of several IL-1/Toll receptor family proteins and mediates their intracellular responses [18], [19]. Since swapping the TIR domain of IL1RAPL1 with that of IL-1R1 abolished the stimulatory effect on the number of dendritic protrusions [14], we focused onthe possible role of Mcf2l in IL1RAPL1-mediated synapse formation.

**Figure 6:**
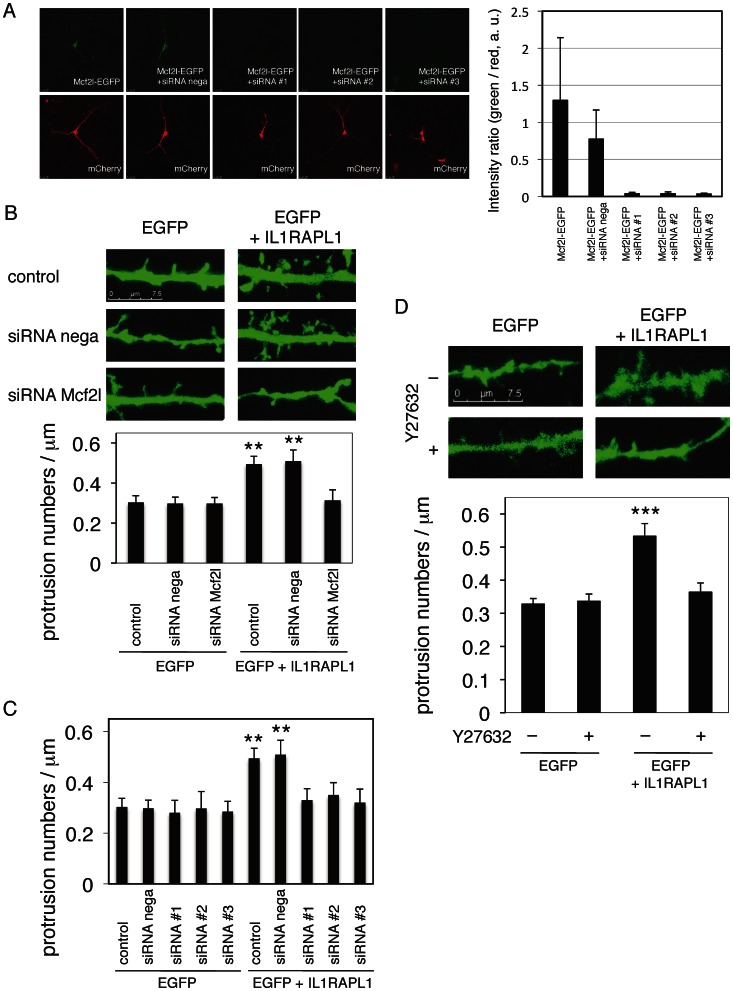
Mcf2l and ROCK regulate IL1RAPL1-induced dendritic protrusion numbers in cortical neurons. **A,** Test of siRNA knock-down efficiency for Mcf2l in cultured cortical neurons. Mcf2l-EGFP and mCherry were cotransfected with siRNAs. Representative patterns of Mcf2l-EGFP and mCherry expression are shown (left). The ratios of fluorescence intensities of Mcf2l-EGFP (green) to mCherry (red) were calculated (right, *n* = 5, *n* = 3, *n* = 5, *n* = 5, *n* = 7 [left to right bars]). **B,** Effects of endogenous Mcf2l knock-down on IL1RAPL1-mediated increase of protrusion numbers along the dendrites. Typical images are shown as representative (top). Six bars [EGFP]: *n* = 16, *n* = 10, *n* = 13, [EGFP+IL1RAPL1]: *n* = 10, *n* = 4, *n* = 11, *F* = 4.92, *p*<0.001, ANOVA (bottom). **C,** Three independent siRNA knock-down experiments for endogenous Mcf2l on IL1RAPL1-mediated increase of protrusion numbers along the dendrites. Ten bars [EGFP]: *n* = 16, *n* = 10, *n* = 5, *n* = 5, *n* = 5, [EGFP+IL1RAPL1]: *n* = 10, *n* = 4, *n* = 5, *n* = 5, *n* = 5, *p*<0.001, ANOVA (bottom). **D,** Effects of Y27632 on IL1RAPL1-mediated increase of protrusion numbers along the dendrites. Typical images are shown as representative (top). Four bars [EGFP]: *n* = 27, *n* = 32, [EGFP+IL1RAPL1]: *n* = 32, *n* = 25, *F* = 12.5, *p*<0.0001, ANOVA (bottom). **, *p*<0.01; ***, *p*<0.001. Error bars represent s.e.m.

First, siRNAs were designed for Mcf2l using the BLOCK-iT RNAi Designer (Invitrogen). Six top-scored siRNA target sequences (Stealth RNAi) were synthesized and their effects on the expression of Mcf2l-EGFP were tested in cotransfected HEK 293T cells. Three siRNAs (#1, #2 and #3) selected strongly suppressed the expression of Mcf2l-EGFP in cultured cortical neurons (Figure 6A). Treatment of cortical neurons with siRNA hardly affected the numbers of dendritic protrusions but diminished the IL1RAPL1-dependent increase of dendritic protrusion numbers (Figure 6B). Three independent siRNAs against Mcf2l showed suppressive effects on the increase of dendritic protrusion induced by IL1RAPL1 (Figure 6C). These results suggest that knock-down of endogenous Mcf2l by siRNA suppressed the IL1RAPL1-mediated increase of dendritic protrusions in cortical neurons.

Mcf2l (also known as Dbs or Ost) is a Rho guanine nucleotide exchange factor (GEF) that activates RhoA and Cdc42 [20]. RhoA and Rac1 have been implicated in the cytoskeletal dynamics that induce structural change of excitatory spines [21], [22] and actin cytoskeletal dynamics are regulated by RhoA-dependent activation of ROCK [23], [24]. We thus examined the effect of ROCK inhibitor Y27632 on the IL1RAPL1-mediated synapse formation. Treatment of cortical neurons with 10 μM Y27632 hardly affected the numbers of dendritic protrusions but suppressed IL1RAPL1-induced protrusion formation of cortical neurons (Figure 6D). These results suggest that IL1RAPL1 regulates the dendritic protrusion formation through ROCK signaling pathway.

**Figure 7:**
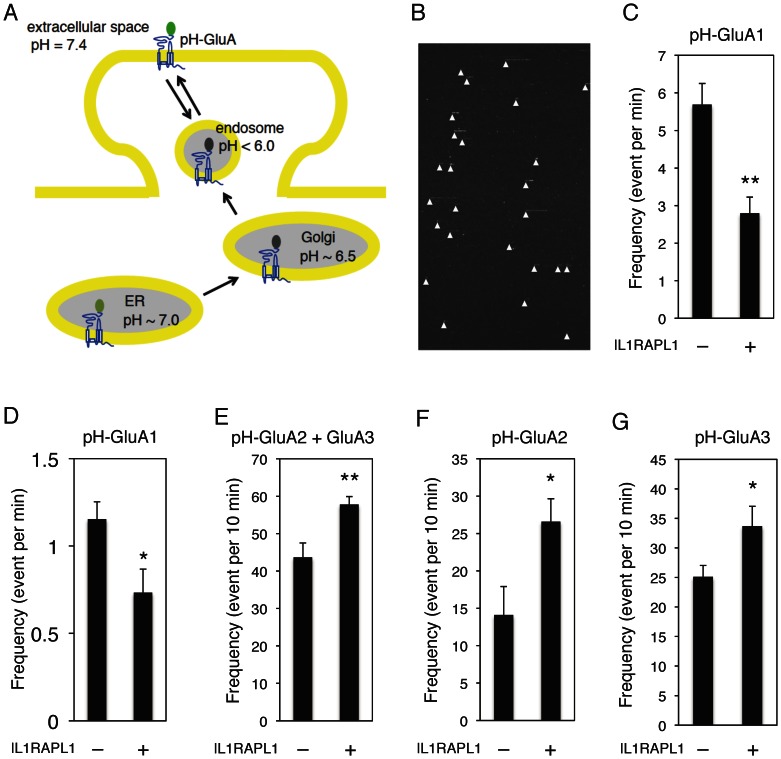
IL1RAPL1 regulates AMPA receptor newly insertion to surface in cortical neurons. **A,** pHluorin fluorescence of pH-GluA in neurons. pHluorin signals are invisible in Golgi and endosome (in low pH) and weakly visible in the endoplasmic reticulum (ER, pH ∼7.0). Bright punctate signals of fluorescence increase when pH-GluA is inserted to surface and the pHluorin tag is exposed to the extracellular space (pH 7.4). **B,** Representative real time visualization of typical pH-GluA1 insertion events. Signal position around a neuron (y-axis, 83 μm) and time (x-axis, 5 min). Each ‘comet-like’ event is indicated by a white arrowhead. The sudden rising and disappear in fluorescence represents individual surface expression of pH-GluA1. **C, E–G,** Effects of IL1RAPL1 overexpression on the insertion frequency of pH-GluA1 (*n* = 9, *n* = 7) (**C**), pH-GluA2/GluA3 (*n* = 10, respectively) (**E**), pH-GluA2 (*n* = 10, respectively) (**F**) and pH-GluA3 (*n* = 10, respectively) (**G**). **D,** Longer observation of IL1RAPL1 effects on the pH-GluA1 insertion frequency (*n* = 4, respectively). Signals existing on surface over 1 min were calculated. Student *t*-test. *, *p*<0.05; **, *p*<0.01. Error bars represent s.e.m.

The expression level of postsynaptic α-amino-3-hydroxy-5-methyl-4-isoxazole propionate (AMPA)-type glutamate receptors in dendritic spines is proportional to excitatory synaptic functions [25], [26]. We thus examined the AMPA receptor trafficking events by measuring the frequency of individual AMPA receptor newly insertion to surface in cortical neurons. An established method with total internal reflection fluorescence microscopy (TIRFM) was used to image the fluorescence signal of superecliptic pH-sensitive GFP (pHluorin)-tagged GluA1 (pH-GluA1), pH-GluA2 and pH-GluA3 in cultured cortical neurons. Bright punctate pHluorin signals would be visible only in the extracellular space when pHluorin-tagged AMPA receptors were inserted to the plasma membrane in this condition (Figure 7A) [27]-[29]; pH-GluA1, pH-GluA2 and pH-GluA3 were shown to be present at synapses in cultured neurons [29], [30]. By imaging pHluorin under TIRFM, we are able to visualize the appearance of surface pH-GluA1/2/3 (an typical example of real observation of pH-GluA insertion events is shown in Video S1). Then, time rendering images were generated, respectively (the same image with Video S1 are converted to Video S2). An example of generated images used in these analyses is shown (Figure 7B from Video S1 and Video S2). X-axis means time course and y-axis stands for pH-GluA signal position around a neuron in this image. Each ‘comet-like’ event in the image indicates surface expression of individual pH-GluA and white arrowheads show the start points of pH-GluA insertion. Our live imaging results showed that the expression of IL1RAPL1 for 2 to 3 days reduced the newly insertion frequency of pH-GluA1 (Figure 7C and Figure 7D). In contrast, insertion rates of pH-GluA2/GluA3 (Figure 7E), pH-GluA2 (Figure 7F) and pH-GluA3 (Figure 7G) were increased by IL1RAPL1 under basal condition, which corresponded with the IL1RAPL1-induced increase of spine numbers [14]. These data indicate the changes of newly inserted AMPA receptor composition during the expression of IL1RAPL1 for 2 to 3 days. Replacement of AMPA receptors from the initially inserted GluA1-containing receptor to the constitutive recycling GluA2/3 receptor may reflect the conversion from newly formed synapses to stably existing excitatory synapses.

**Figure 8:**
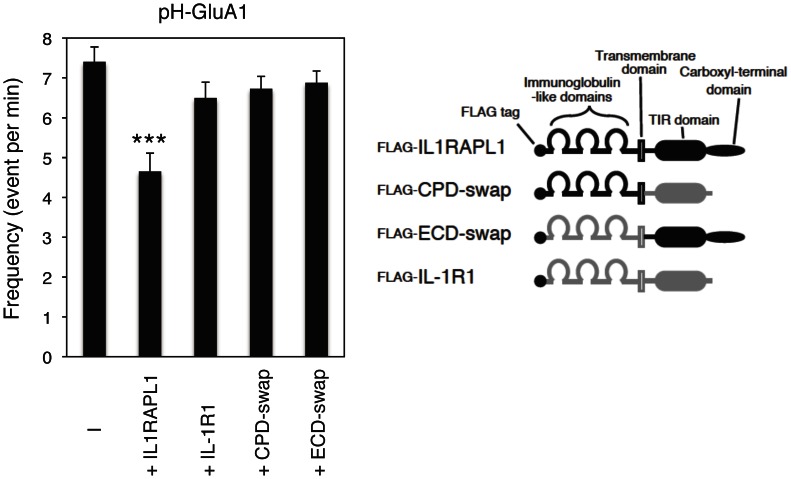
Effects of IL-1R1 and IL-1R1/IL1RAPL1 chimeras overexpression on pH-GluA1 insertion. Schematic structures of FLAG-IL1RAPL1, FLAG-IL-1R1 and their swap mutants are shown. The frequency of pH-GluA1 insertion was measured in cortical neurons transfected with IL1RAPL1, IL-1R1 or their swap mutants, *n* = 8, respectively, *F* = 7.98, *p*<0.0001, ANOVA). ***, *p*<0.001. Error bars represent s.e.m.

We also examined the effects of domain swap mutants between IL1RAPL1 and IL-1R1 [14] on the insertion of pH-GluA1 to cell membrane. IL-1R1 and extracellular or intracellular domain swap mutants between IL1RAPL1 and IL-1R1 failed to mimic IL1RAPL1 in TIRFM assay for the insertion of pHluorin-tagged AMPA receptors to the plasma membrane (Figure 8). These results are consistent with our previous observation that these swap mutants and IL-1R1 failed to stimulate the formation of dendritic protrusions in cortical neurons [14].

**Figure 9:**
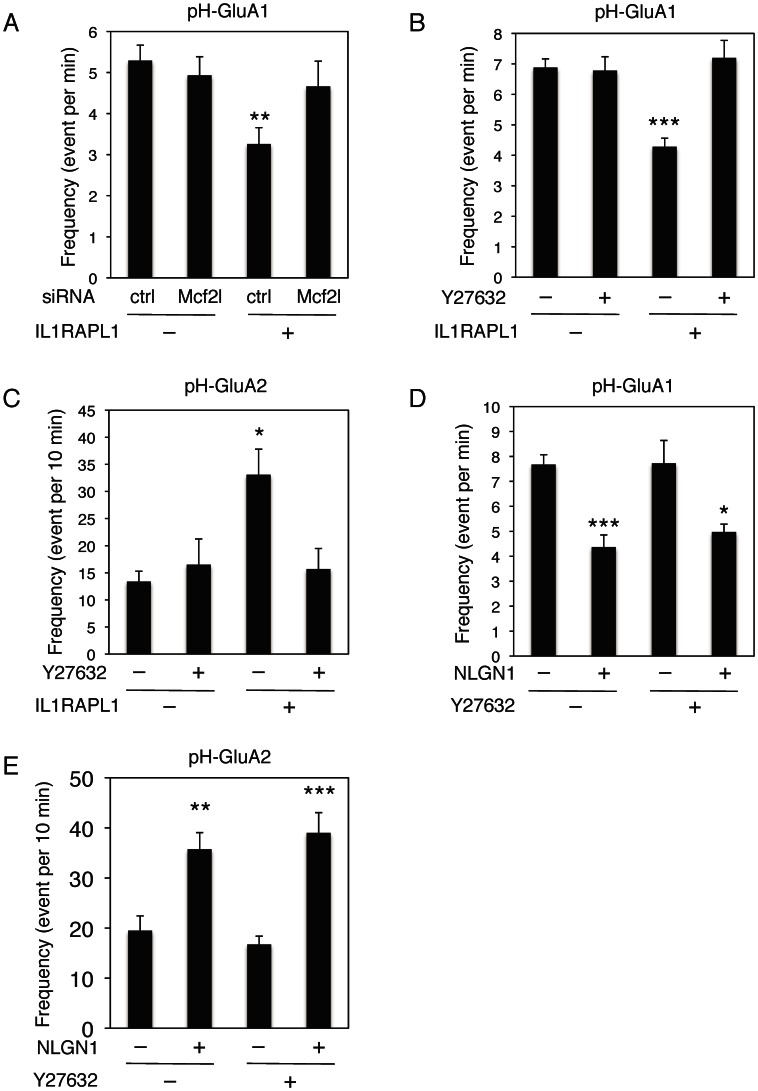
RhoA signaling pathway regulates IL1RAPL1-mediated AMPA receptor newly insertion to surface in cultured cortical neurons. **A,** Effects of knock-down of endogenous Mcf2l on IL1RAPL1-mediated suppression of pH-GluA1 insertion (*n* = 9, *n* = 9, *n* = 10, *n* = 10, *F* = 3.54, *p*<0.05, ANOVA). **B,** Effects of Y27632 on IL1RAPL1-mediated suppression of pH-GluA1 insertion (*n* = 10, respectively, *F* = 10.4, *p*<0.0001, ANOVA). **C,** Effects of Y27632 on IL1RAPL1-induced increase of pH-GluA2 insertion (*n* = 10, respectively, *F* = 5.21, *p*<0.005, ANOVA). **D,** Influences of NLGN1 overexpression on pH-GluA1 insertion with or without Y27632 (*n* = 8, respectively, *F* = 9.36, *p*<0.0002, ANOVA). E, Influences of NLGN1 overexpression on pH-GluA2 insertion with or without Y27632 (*n* = 8, respectively, *F* = 13.1, *p*<0.0001, ANOVA). Cultured cortical neurons were treated with 10 μM Y27632 for 3 days. *, *p*<0.05; **, *p*<0.01; ***, *p*<0.001. Error bars represent s.e.m.

Finally, we examined the blockade of the RhoA signaling pathway in IL1RAPL1-dependent changes of AMPA receptor insertion. Our data showed that siRNA knock-down of endogenous Mcf2l expression suppressed IL1RAPL1-dependent decrease of pH-GluA1 insertion to surface (Figure 9A). Moreover, treatment with ROCK inhibitor Y27632 (10 μM) eliminated the effect of IL1RAPL1 on pH-GluA1 insertion (Figure 9B). IL1RAPL1-dependent increase of pH-GluA2 insertion was reduced by the treatment with Y27632 (Figure 9C). Similar to IL1RAPL1, the expression of neuroligin 1 (NLGN1) reduced pH-GluA1 insertion (Figure 9D) and enhanced pH-GluA2 insertion (Figure 9E). However, treatment with Y27632 hardly affected these NLGN1-dependent alterations of AMPA receptor insertion (Figure 9D and Figure 9E). These results suggest that Mcf2l-RhoA-ROCK signaling pathway functions in the downstream of IL1RAPL1 in excitatory synapse stabilization.

## Discussion

We previously found that postsynaptic IL1RAPL1 mediates excitatory synapse formation through *trans*-synaptic interaction with presynaptic PTPδ in mouse cortical neurons and have proposed that the impairment of synapse formation underlies certain forms of MR and ASD as a common pathogenic pathway [14]. IL1RAPL1 stimulates the increase of dendritic protrusions through the TIR domain [14]. To examine the signaling pathway through which IL1RAPL1 regulates synapse formation, we here identified Mcf2l and PKCε as TIR domain-binding proteins. Since PKCε interacts with the TIR domains of many IL-1/Toll receptor family proteins [18], [19], we focused on Mcf2l. We showed that siRNA-mediated knockdown of Mcf2l and ROCK kinase inhibitor treatment suppressed IL1RAPL1-induced spinogenesis in cultured cortical neurons. These results suggest that IL1RAPL1 controls spine formation of cortical neurons through Mcf2l-RhoA-ROCK signaling pathway. Since IL1RAPL1-induced increase of dendritic protrusions requires presynaptic PTPδ [14], *trans*-synaptic interaction between IL1RAPL1 and PTPδ may affect RhoGEF activity of Mcf2l to sitmulate spinogenesis. Treatment of cortical neurons with siRNAs against Mcf2l or a ROCK inhibitor hardly affected the number of dendritic protrusions but diminished the IL1RAPL1-mediated increase of dendritic protrusions. It is possible that IL1RAPL1-induced spinogenesis is mediated by multiple signaling pathways in cortical neurons. Other pathway(s) besides Mcf2l-RhoA-ROCK pathway may support the endogenous IL1RAPL1-mediated spinogenesis when Mcf2l-RhoA-ROCK pathway in cortical neurons was suppressed by treatment with siRNAs against Mcf2l or a ROCK inhibitor. When IL1RAPL1 was overexpressed in cortical neurons, however, these signaling molecules may be fully utilized and the effects of these treatments became evident. Understanding of the regulatory mechanism of central synapse formation will be important for providing molecular targets for drug intervention and treatment of human neurological disorders. Interestingly with this respect, it is to be noted that Mcf2 RhoGEF, a Mcf2l homolog, and small GTPases including Ras and Rho are closely related to MR and ASD [31], [32].

We also identified PLCβ1, SNIP and Rasal1 as CT domain-binding proteins and found actin-binding cytoskeletal/scaffolding protein spectrin α2 and β2 as possible IL1RAPL1-interacting proteins. It is possible that IL1RAPL1-induced spinogenesis might be mediated by some of these molecules. However, pathophysiological significance of these interactions remains to be examined. It is reported that the CT domain of IL1RAPL1 interacts with the neuronal calcium sensor-1 and postsynaptic density protein 95 (PSD-95) [33]–[35] and that RhoGAP2 is associated with the ICD of IL1RAPL1 [36]. However, these molecules were not found in our screening by affinity chromatography. IL1RAPL1 was reported to regulate N-type voltage-gated calcium channel and neurite elongation in PC12 cells through the neuronal calcium sensor-1 and to control the synaptic localization of PSD-95 by regulating c-Jun terminal kinase activity [33]–[35].

Previous studies revealed the rules for subunit-specific AMPA receptor trafficking [37]. Initially, synaptic delivery of GluA1-containing AMPA receptor occurs in activity- or experience-dependent manner. Constitutive replacement of synaptic GluA1 by GluA2/3 follows and takes up to 20 hr [38]. This process may be important to maintain synaptic strength unchanged and may stabilize recently formed synapses [37]. Novel protocols to observe the replacement of AMPA receptor composition with TIRFM enable us to visualize the synaptic stabilization after the formation of excitatory synaptic connections. While it is hard to distinguish GluA1/2- and GluA2/3-functional AMPA currents electrophysiologically, TIRFM imaging allows to follow the subunit-specific insertion event. Our experiments using pH-GluAs showed that the expression of IL1RAPL1 for 2 to 3 days led to the replacement of newly inserted AMPA receptor compositions in cortical neurons through the Mcf2l-RhoA-ROCK signaling pathway. Mcf2l-RhoA-ROCK-dependent cytoskeletal dynamics may be involved in the formation of stable excitatory spines in the cortex. IL1RAPL1 should induce new functional excitatory synapses since IL1RAPL1 knockout reduced miniature EPSC frequency with little effect on amplitude and its overexpression increased the frequency [35]. Thus, the IL1RAPL1-Mcf2l-RhoA-ROCK signaling pathway mediates functional formation of glutamatergic synapses. NLGN1, which regulates the maturation of excitatory synapses [39], also influenced AMPA receptor trafficking, but in a manner independent of the RhoA signaling pathway. In summary, we revealed that IL1RAPL1 regulates the formation and stabilization of glutamatergic synapses between cortical neurons through Mcf2l-RhoA-ROCK signaling pathway.

## Materials and Methods

### Construction of Expression Vectors

The cDNA encoding entire cytoplasmic domain (amino acid residues 379–696) of mouse IL1RAPL1 was amplified with primers, 5′-TGAATTCAAATGTTACAAGATAGAAATCATGC-3′ and 5′-TGCGGCCGCTCACCAGATCACACTGGATATAC-3′ using pIL1RAPL1 [14] as a template and cloned into pCRII-TOPO vector (Invitrogen) to yield pCRII-mIL1RAPL1-Cyto. The 1.0-kb *Eco*RI fragment from pCRII-mIL1RAPL1-Cyto was ligated with the 6.7-kb *Eco*RI fragment from pMAL-c2 vector (New England BioLabs) to yield pMAL-c2-mIL1RAPL1-Cyto.

### Affinity Column Chromatography and Mass Spectral Analyses

BL21 cells (Stratagene) were transformed with pMal-c2 and pMAL-c2-mIL1RAPL1-Cyto and grown at 30°C until log phase before induction with 1 mM isopropyl-β-D-thiogalactopyranoside. Cells were harvested, resuspended in 0.01 culture volume with PBS, and lysed in PBS-0.1% Tween-20 containing protease inhibitor cocktail (Roche). After sonication (10×30 s), cell lysates were centrifuged, and the resulting supernatants were bound to an amylose resin (New England BioLabs). The MBP and MBP-IL1RAPL1-bound amylose resins were then used to pack affinity columns. Mouse brain extracts were loaded onto the columns. The proteins bound to the affinity columns were eluted by the addition of buffer containing 500 mM NaCl. The elutes were subjected to SDS-PAGE, and the bound proteins were evaluated by negative staining. Bands were cut and subjected to in-gel reduction, alkylation and digestion with trypsin followed by analysis by LC-MS/MS as previously described [14], [40].

### Coimmunoprecipitation

HEK 293T cells were transfected with pcDNA3-YFP-IL1RAPL1 or pcDNA3-FLAG-IL1RAPL1 and pcDNA3-FLAG-Bat3, pcDNA3-myc-PLCβ1, pcDNA3-FLAG-Snap91, pcDNA3-myc-SNIP, pcDNA3-FLAG-Mcf2l, pcDNA3-FLAG-Rasal1 or pcDNA3-FLAG-PKCε (CMV promoter) using NanoJuice (Novagen). Cells were lysed in TNE buffer (containing 50 mM Tris–HCl [pH 7.5], 1% Nonidet P-40, 150 mM NaCl, 5 mM EDTA, 1 mM NaF, 1 mM Na_3_VO_4_, 100 μg/ml phenylmethylsulfonyl fluoride (PMSF), 2 μg/ml aprotinin, 0.5 μg/ml leupeptin, and 1 μg/ml pepstatin) and immunoprecipitated with anti-GFP (Molecular Probes, 3E6) or anti-FLAG (Sigma, M2) antibody. Immunoprecipitated samples and cell lysates were separated by SDS-PAGE followed by western blotting with anti-FLAG (Sigma, M2) or anti-Myc (Santa Cruz, 9E10) antibody.

### Immunocytochemistry

Expression vectors for IL1RAPL1 and myc-tagged candidate proteins were transfected to HEK 293T cells using FuGene6 transfection reagent (Roche). After 2 days of culture, the transfected cells were immunostained with goat anti-IL1RAPL1 (R&D Systems) and mouse anti-Myc (Santa Cruz) antibodies followed by incubation with Alexa488-conjugated donkey anti-goat IgG and Alexa555-conjugated donkey anti-mouse IgG antibodies (Invitrogen). The immunostained cells were imaged with a confocal laser-scanning microscope (TCS SP5, Leica; zoom setting, 5; z step, 1–2 μm) using Leica 63× water lens (numerical aperture, 1.20).

### Neuronal Culture and Transfection

For TIRFM, cortical neurons from embryonic day 16 (E16) ICR mice (CLEA Japan, Inc.) were seeded on glass-bottom 35-mm dishes that were precoated with poly-L-lysine (MatTek). The cells were plated in Neurobasal A media (Gibco) containing 50 U/mL penicillin, 50 mg/mL streptomycin and 2 mM glutamine, supplemented with 2% B27 (Gibco) and 5% fetal calf serum (Gibco). Media were switched to feeding media (plating media without serum) at 24 h after plating and maintained them in serum-free condition thereafter to prevent glial cell growth on the surface of glass, which interferes with TIRF microscopy. Then, neurons were subsequently fed twice a week by changing half volume of the feeding media. At 12 days *in vitro* (DIV), cortical neurons were transfected with pRK5-pH-GluA1, pcDNA3.1-pH-GluA2, pRK5-GluA3, pIL1RAPL1, pFLAG-IL-1R1, pFLAG-CPD-swap, pFLAG-ECD-swap, pFLAG-NLGN1 and siRNAs using Lipofectamin 2000 (Invitrogen). Neurons between the ages of 14–16 DIV were used for imaging experiments.

### RNAi

The oligo sequences were 5′-CGGACAAGGAGTTCCAGAATGTCAT-3′ for Mcf2l siRNA #1, 5′-CAGCGTGGGTGAATGAGATTCGGAA-3′ for Mcf2l siRNA #2, 5′-GGAAGGCTACGTGAGCTCATCGTTA-3′ for Mcf2l siRNA #3.

### TIRFM Imaging

The TIRFM imaging system was based on a IX81N-ZDC2-1 microscope (Olympus). The excitation laser was a 488 nm-20 mW (Olympus). The laser was coupled to a TIRF slider via FV5-FUR fiber optics (Olympus). A DM505 dichroic mirror (Olympus) was used to reflect the incoming laser onto a UAPON 100× OTIRF objective (N.A. = 1.49, Olympus). A BP510–550 emission filter was used for pHluorin fluorescence detection (Olympus). An EMCCD camera (ImagEM C9100-13; Hamamatsu Photonics) was used as detector. To detect dim signals, the EMCCD gain was set to maximal. The camera was maintained at –65°C. An Unblitz LS6ZM2 shutter controlled by VMM-D3J (Vincent Associates) was integrated between the laser head and the fiber launcher to control the laser. Data were acquired using Metamorph software (Universal Imaging Co.). All of the imaging experiments were carried out in artificial cerebrospinal fluid (ACSF, 119 mM NaCl, 2.5 mM KCl, 2 mM CaCl_2_, 1 mM MgCl_2_, 25 mM Hepes (pH 7.4) and 30 mM D-glucose) at room temperature. Live cell images were captured every 1 sec for 5 min (pH-GluA1, 300 frames) or every 5 sec for 10 min (pH-GluA2 and pH-GluA3, 120 frames) to generate each movie. We also imaged pH-GluA1 insertion for longer time (every 10 sec for more than 30 min, only for Figure 7D). To increase the signal-to-noise ratio, we typically performed more than 1 min photobleach of preexisting surface AMPA receptors before data acquisition. Recordings were analyzed using Metamorph and insertion events lasting over 5 frames (longer than 5 sec) or over 6 frames (longer than 1 min, only for Figure 7D) for pH-GluA1 and over two frames (longer than 10 sec) for pH-GluA2 and pH-GluA3 were registered as events manually. A typical image is shown as representative (Video S1). Y-t rendering images were generated by rotating the original xyt stack 90° along the y-axis using maximum intensity projection algorithm (Video S2). Total events per minute were taken as the frequency of pH-GluA insertion (Figure 7B). Individual experiments were performed using sister cultures. pH-GluA insertion rates obtained were in good agreement with the values reported previously [27]–[29].

### Statistics

All of the statistical tests were performed using Excel (Microsoft). Values were expressed as mean ± s.e.m. Comparisons for two groups of data were done by two-tailed Student’s *t*-test. Multiple comparisons were done by one-way ANOVA followed by Tukey posthoc test.

## Supporting Information

Video S1 **Real time visualization of typical pH-GluA1 insertion events.** pHluorin-tagged GluA1 was transfected in cortical neurons and insertion events were observed at DIV14-16 by TIRF microscopy (scale bar, 20 μm).
(MPG)

Video S2 **Y-t rendering images.** Images were generated by rotating the original xyt stack 90° along the y-axis, and the maximum intensity of each x-line was projected onto a single pixel of the y-axis using a maximum-intensity projection algorithm.
(MPG)
